# A Power Planning Algorithm Based on RPL for AMI Wireless Sensor Networks

**DOI:** 10.3390/s17040679

**Published:** 2017-03-25

**Authors:** Marcio L. F. Miguel, Edgard Jamhour, Marcelo E. Pellenz, Manoel C. Penna

**Affiliations:** PPGIa, Pontifical Catholic University of Parana—Parana, Curitiba 80215-901, Brazil; marcio.miguel@gmail.com (M.L.F.M.); jamhour@ppgia.pucpr.br (E.J.); marcelo@ppgia.pucpr.br (M.E.P.)

**Keywords:** smart grid, advanced metering infrastructure, low power and lossy networks, neighborhood area network, destination oriented acyclic graph

## Abstract

The advanced metering infrastructure (AMI) is an architecture for two-way communication between electric, gas and water meters and city utilities. The AMI network is a wireless sensor network that provides communication for metering devices in the neighborhood area of the smart grid. Recently, the applicability of a routing protocol for low-power and lossy networks (RPL) has been considered in AMI networks. Some studies in the literature have pointed out problems with RPL, including sub-optimal path selection and instability. In this paper, we defend the viewpoint that careful planning of the transmission power in wireless RPL networks can significantly reduce the pointed problems. This paper presents a method for planning the transmission power in order to assure that, after convergence, the size of the parent set of the RPL nodes is as close as possible to a predefined size. Another important feature is that all nodes in the parent set offer connectivity through links of similar quality.

## 1. Introduction

Advanced Metering Infrastructure (AMI) is an architecture for two-way communication between smart utility meters and utility companies. The goal of AMI in the smart grid is to provide utility companies with real-time data about power consumption and allow customers to make informed choices about energy usage based on the price at the time of use. The smart grid includes many other features in addition to measuring energy consumption. The AMI network can provide bidirectional communication for services like distributed power generation, demand side management for customer load control in the customer’s premises, and disconnections/re-connections of users due non-payment, among others.

An important part of the AMI is communication in the neighborhood area, which usually involves a lot of communication nodes deployed in a large and complex geographical area. Accordingly, the communication network has to cover all metering devices, providing connections to all nodes. Considering the requirements of complexity and scalability of AMI, it is well accepted that wireless communication offers a larger degree of freedom for information collection and actuation over metering devices.

In this study, we refer to an AMI network as the wireless sensor network that provides two-way communication for metering devices in the neighborhood area of the smart grid. The AMI network is structured in the sense that the positions of its nodes are fixed and known. Communicating nodes deployed in the AMI network have limited resources, that is, they use micro-controllers with low processing power and limited memory capacity. In addition, they are powered by the electric grid, which implies that there are not considerable concerns about power consumption, which could be a problem for battery-powered devices. Although some countries define in their local regulations the maximum power that is available to smart meters and connected communication devices, the defined limits are sufficient for most cases. However, as in most wireless sensor networks, devices are interconnected by unreliable links of low-bandwidth. These requirements are considered by the Internet Engineering Task Force (IETF) in the standardization of the routing protocol for low power and lossy networks (RPL) [1], which propose a routing protocol compatible with IPv6. Because RPL is designed for lossy networks, it is being considered as an important option for AMI network deployment [2,3,4,5,6]. As it operates in the unlicensed 915 MHz band, interferences from external devices may be present. However, the RPL is designed to react to changes in transmission conditions, and it is capable of mitigating possible interference problems coming from other electronic devices.

RPL builds a routing topology formed by one or more destination oriented acyclic graphs (DODAG). Each DODAG is rooted in a single destination, usually representing a node that performs a connection to a back-haul. A DODAG differs from a traditional tree because it permits a node to have more than one parent in the direction of the root. RPL uses a “slow” proactive process to construct and maintain a routing topology, and a reactive and dynamic process to resolve route inconsistencies [7,8,9]. RPL uses the “trickle algorithm” [10] that determines that, in steady state, the protocol control messages are sent at a slow rate, but can be quickly increased to solve inconsistencies. The standard introduces the concept of RPL instance to allow different routing criteria to be considered on the same network. An RPL instance has an objective function that defines how the routing metric selects the parents of a node in a DODAG. The objective function computes a rank value, which represents the distance from the node up to the root with respect to a given metric.

The expected transmission count (ETX) [4] is one of the most relevant metrics in wireless networks because it dynamically evaluates the quality of the communication link perceived by each node. Obviously, the construction of DODAG will be heavily dependent on ETX when it is the metric used in the objective function. However, RPL does not define any protocol to measure ETX. A common approach is to measure it using information from the media access control (MAC) layer, as implemented by the Contiki operating system [11,12]. The MAC-based quality estimation of a link can be used only when unicast data traffic is sent through this link. Because RPL builds the topology using multicast control messages, the DODAG should be built using default initial values for ETX [12]. This leads to the use of bad quality links, which can have numerous consequences (see Section 2).

Another important point comes from the size of parent sets. A small parent set can cause instability to the DODAG if the quality of the links are highly variable. When a node identifies that it is not connected to any parent, it sends a DODAG Information Solicitation (DIS) message to ask for new parents, causing the reconstruction of the sub-tree below it. A large parent set can also cause problems to RPL because the rank of a node must be greater than the rank of any member of its parent set. Even though a node should select the best node in the parent set as the preferred parent, it computes its rank and the cost of the path to the root considering the worst element in the parent set. Thus, using an excessively large parent set may inhibit a child node to select the best parent and will likely result in sub-optimal routing [13]. There is a tradeoff concerning the size of parent sets, which can be neither too small nor too large. Although it is an important parameter for the resilience of the DODAG, RPL only allows the maximum number of parents to be defined. If a certain amount of parents is needed to meet resilience requirements, RPL does not have mechanisms to pursue this goal. To address this issue, we propose a power adjustment method, which can be used to plan DODAGs with predefined size for the sets of parents, without compromising the quality of the paths to the root.

Nodes in wireless sensor networks can adjust their transmission power to increase the network connectivity. However, using the highest power level for all nodes can result in a significant reduction in average lifetime and capacity of the network. Because the nodes in AMI networks are not battery powered, the first issue does not arise, but the second should be addressed. In this sense, to improve the DODAG connectivity and resilience, we propose an algorithm that assigns power levels to the transmitters in RPL networks.

Power level assignment selects the transmission power so that the resulting communication graph respects certain constraints and minimizes energy consumption. Kirousis et al. [14] proved that this problem is NP-hard for sets of three-dimensional points, and Clementi et al. [15] proved the same for sets of two-dimensional points. Carmi and Katz [16] considered the problem for the case where each node can transmit in one of two possible power levels. They proved that this problem is also NP-hard, and provided two heuristic algorithms to solve it. Sisodiya and Shetty [17] provide an almost optimal heuristic for the same case, which aims to minimize total power consumption while maintaining a strong level of connectivity. They also present a heuristic algorithm to minimize total power using three power levels. Lloyd et al. [18] studied the problem for the case where nodes can transmit at any power level. They provided an approximation algorithm for minimizing the total power for some monotone properties, which is not realistic because there is a limited amount of transmission levels. More recently, a few studies have attempted to determine the optimal set of power levels. Zou et al. [19] defined the optimal probability distribution associated with these sets, while Tantur et al. [20] investigated the minimum cardinality that does not compromise the service life of the WSN. Differently, in this paper, we present an RPL-based heuristic approach for transmission power level assignment.

Most of the RPL evaluations that motivated the previous critics paid little attention to the configuration of the transmission power of the RPL nodes. However, adjusting transmission power is important in AMI networks because they present strong performance requirements in terms of two-way communication, and the adjustment could assure the quality of every bidirectional link. In addition, power planning allows the search for optimal connectivity in terms of the number of alternative parents in the DODAG. Power planning is feasible in AMI networks because their nodes do not have strict energy restriction, and it can be executed offline because the positions of nodes are fixed and well known.

This paper presents a study about the planning of transmission power of the nodes in AMI networks, in particular those based on the IEEE 802.15.4 technology. We defend the viewpoint that a careful planning of the transmission power of the communication nodes can significantly reduce the problems raised for RPL. More precisely, this paper presents a method for planning the transmission power that assures that the size of the parent set of the RPL nodes is as close as possible to a predefined size. Another important feature of the proposed method is that the resulting power setting allows all nodes in the parent set to offer connectivity through links of similar quality. The consequence is that a node is capable of making more realistic path cost offers for their children.

We evaluate our algorithm considering the ability of RPL to find reliable parent sets under different situations, such as highly concentrated urban and sparse rural areas. As far as we know, this is the first power-planning algorithm proposed for RPL-based networks.

The remaining of this paper is organized as follows: Section 2 details the problem and gives some examples that motivate the use of a power planning algorithm. Section 3 presents a theoretical model to estimate the ETX metric. We use the theoretically estimated ETX to compute the transmission power of the nodes and to give an initialization value for the DODAG construction. In Section 4, we describe the algorithm, showing how the transmission power of nodes and of their parents are adjusted to assure that all links have the ETX below a given threshold. Section 5 evaluates the algorithm, comparing the results with the default power setting and initial values of ETX used in most RPL evaluations based on Cooja/Contiki. Finally, Section 6 presents the final remarks and concludes the paper.

## 2. Problem Statement

During the formation of DODAG, each node should select a set of nodes eligible as parents (the parent set), select its preferred parent, as well as calculate and propagate its rank. A node takes these decisions each time it receives an offer of a path to the root node (a DIO message). The candidate nodes to be included in the parent set are those from which a node receives a DIO message with a value of rank that is lower or equal than the current rank of the node. The cost of the path to the root through a node in the parent set is calculated by adding the rank of that node with the cost of the corresponding link. The preferred parent is the one whose path cost to the root is the lowest.

**Figure 1:**
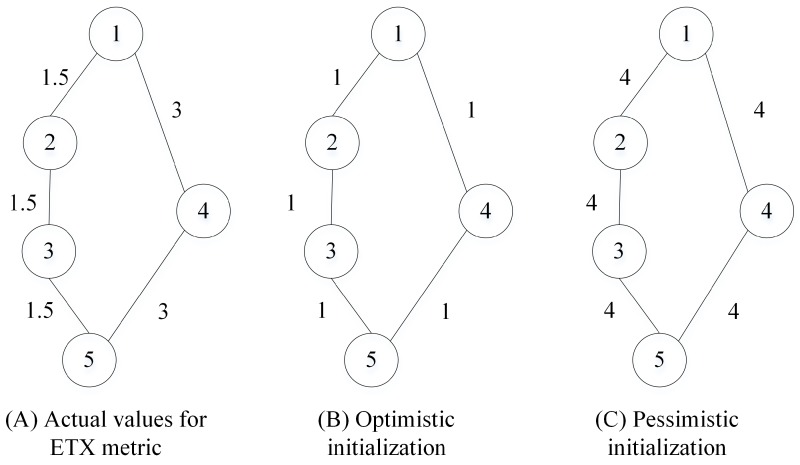
Initialization of expected transmission count (ETX) metric.

RPL specifies the ETX as the metric for the objective function when the quality of the link is critical [13]. However, RPL does not define any protocol to measure ETX. A common approach is to use information from the MAC layer, but the estimation of ETX based on MAC layer information can only be done when unicast data traffic is sent through the links. Because RPL builds the DODAG using multicast control messages, it is forced to build DODAG based on predefined initial values for the ETX. All evaluations of RPL in Contiki referred in this paper start measuring ETX only when the unicast data traffic is actually transmitted. Figure 1 shows an example of a DODAG rooted in node 1, with the values for ETX presented beside the links. If the initial values are optimistic as in Figure 1B, the preferred route for node 5 would be (5,4,1) through bad quality links. This bad choice is only fixed when nodes start sending data and the actual values are computed. In this case, a node tends to keep changing parents until all nodes in the parent set are evaluated and receive a more realistic ETX. If initialization values are pessimistic as in Figure 1C, the rank of every node in the DODAG will be overrated, resulting in suboptimal routing. In this case, a node tends to keep using the first chosen parent, once the actual value for ETX is lower than initialization. Indeed, some performance studies have pointed out that RPL may suffer from unreliability problems as it may select suboptimal paths with highly unreliable links [2,3], incurring a high likelihood of a DODAG fragmentation [6]. In Section 3, we propose a theoretical model which gives an initial estimate for the value of ETX.

**Figure 2:**
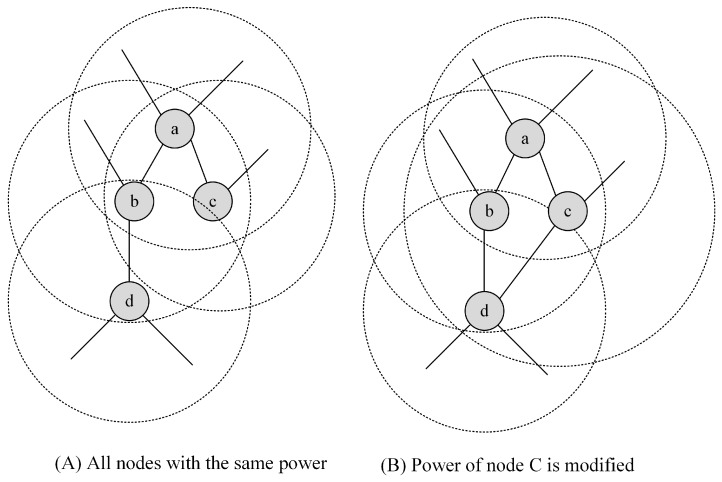
Power planning.

Adjusting the transmission power control can produce DODAGs that are more robust, avoiding disruption and reconfiguration. This is important because reconfiguration is costly in terms of the transmission of control messages and packet loss. Figure 2A shows a part of a DODAG in which all nodes are configured to the same transmission power. Each of the nodes a, b, and c have two members in their parent sets, while d only has one. If node b fails or links (d, b) degrades, the sub-tree below node d is compromised, forcing the protocol to reconfigure the DODAG. In this case, it would be helpful to make a power setting to node c to include the d node in its transmission range, as shown in Figure 2B. Usually, link failures do not trigger global network re-optimization; however, repairs may be very costly when a node detects that its parent set is empty. Once detected, the connectivity failures may cause local (and possibly global) repairs, causing data loss and delivery delays that are incompatible with the AMI requirements. In Section 4, we present a power scheduling algorithm that is capable of inducing RPL to build a DODAG with a number of parents very close to the planned quantity.

## 3. ETX Theoretical Model

RPL supports a flexible set of metrics and constraints [21]. One of the preferred metrics is ETX, which represents the expected number of transmissions required to successfully transmit and acknowledge a packet on a wireless link. The ETX metric was introduced in [4], and is defined by the following expression

(1) $$ETX=\frac{1}{d_{f}*d_{r}},$$

where $d_{f}$ is the measured probability that a packet is received by the neighbor and $d_{r}$ is the measured probability that the acknowledgment packet is successfully received. ETX is sensitive to packet loss but not to delay. If the transmission of a packet is delayed by contention, but the packet is transmitted, the metric is not affected. Thus, the ETX metric is affected by two main components: the quality of the wireless channel and collisions. RPL does not assume a probe mechanism based on broadcast messages, as in [4], to compute the value for ETX. In the following, we present a theoretical model to estimate a value for ETX that is used as the initial value during the DODAG construction.

A channel outage probability model gives a prediction for the packet error rate (PER) caused by radio channel impairments, such as path loss and fading. The Nakagami-*m* distribution [22] is employed in this work to model the multi-path fading effects of the propagation environment. The fading severity is adjusted through the parameter *m*. Supposing the use of capacity achieving codes, an outage occurs at the receiver when the instantaneous signal-to-noise ratio (SNR) γ is below a threshold $\beta =\left(2^{\Delta }-1\right)$ that allows error free decoding. The parameter Δ is the system spectral efficiency. The Nakagami-*m* fading outage probability for frames transmitted from a node with power *w* to a receiver at a distance *d* is

(2) $$O=P\left[\gamma <\beta \right]=\frac{\Psi \left(m,\frac{m\cdot \beta }{\bar{\gamma }}\right)}{\Gamma (m)},$$

where $\bar{\gamma }=\frac{\eta_{d}\cdot w}{N_{0}\cdot B}$ is the average received SNR, *B* is the system bandwidth in Hertz, $\eta_{d}=\frac{G\cdot \lambda^{2}}{{\left(4\pi \right)}^{2}d^{\alpha }}$ is the path loss of the channel and $N_{0}$ is the thermal noise power spectral density in Watts/Hertz. We assume a path loss model with attenuation factor $\frac{1}{d^{\alpha }}$, where α is the path loss exponent used to represent different environment propagation conditions. *G* is the total gain of the transmission and reception antennas, and λ is the wavelength. The Γ(a) and Ψ(a,b) are the complete and lower incomplete gamma functions. The ETX metric of a wireless link between nodes *a* and *b* can be calculated by Equation (3), considering the outage probability of transmissions from *a* to *b* ($O_{ab}$) and *b* to *a* ($O_{ba}$):

(3) $$ETX=\frac{1}{\left(1-O_{ab}\right)*\left(1-O_{ba}\right)}.$$

The ETX of a wireless link can be reduced by increasing the transmission power of the adjacent nodes, but this also increases the interference range caused by this link over other links in the same channel frequency. The interference range can be defined as the distance where the received power equals the receiver carrier-sensing sensitivity.

## 4. DODAG-Oriented Power Planning Algorithm

The proposed power-planning algorithm is designed to be executed offline. An optimal solution to the power assignment in RPL-based networks depends on the objective function. Having multiple parents is a key concept for RPL. A lot of effort is spent to build the DODAG, so it must be as stable as possible. The path cost to the root is another key concept. A lower path cost may be achieved by increasing the transmission power, which also decreases the average rank of the nodes. However, increasing transmission power also increases interference and may unnecessarily decrease the network performance due to excessive contention.

In order to address the number of parents and the path cost issues simultaneously, and propose a solution that is traffic independent, we have designed an algorithm that targets the following property: “find the minimal transmission power configuration that permits to build a DODAG where each node has at least *k* parents accessible with link quality ETX≤Q”.

Finding the optimal solution for DODAG-based power assignment is equivalent to solving the NP-hard problem of power level assignment [14,15], as discussed in the introductory section. Therefore, we adopt a heuristic approach that is based on the logic of the RPL protocol. The following statements are important to understand the heuristics used to design the algorithm:

1. The algorithm assumes that RPL will use the minimum rank with hysteresis objective function (MRHOF) with non-greedy approach [13], i.e., nodes will ignore parent offers from nodes with rank equal or higher than its current rank.
2. When computing the parent set size of a node, the algorithm will consider only parents with very similar path costs, or more precisely, parents that result in the same rank for the node.
3. The power of a node is increased only to the point that it achieves the minimum required number of parents or it can transmit directly to the root.

The first statement is a design choice. Because we are adjusting the transmission power to permit a certain number of parents accessible through links with controlled quality, a node does not need to decrease its rank to accommodate bad positioned parents. This is the non-greedy behavior, one of the solutions recommended to prevent loops [1]. According to RPL, the rank advertised by a node is strongly dependent on the worst element in the parent set [13]. This approach may mask the actual path cost if the elements of the parent set are too diverse. Therefore, it may lead to sub-optimal choice of routes, which is one of most severe critiques addressed regarding RPL. The second statement is a design choice that minimizes the selection of sub-optimal routes. It requires that parents who induce a higher rank be removed from the parent set, when the algorithm finds a parent that reduces the the rank of the node. Therefore, increasing the power of a node does not necessarily increase the number of parents. The third statement is a direct consequence of this condition. Please refer to Table 1 for a description of the symbols used in the remainder of this section.

To perform the power adjustment, the algorithm defines two important metrics related to the DODAG: the path cost from node *n* to the root through parent *i*:

(4) $$\mathrm{cost}\left(n,i\right)=r_{i}+m*etx_{n,i}$$

and the calculated rank of node *n* through parent *i*:

(5) $$\mathrm{rank}\left(n,i\right)=r_{i}+\mathrm{Floor}\left(1+m*etx_{n,i}/h\right)*h,$$

where $r_{i}$, represents the absolute rank of the node, and $etx_{n,i}$ is the ETX of the link between nodes *n* and *i*. The Floor operation and the parameters *h* and *m* are defined in RPL to represent the rank and ETX by unsigned integer values with 16 bits. The default value of *h* is 256 while the default value of *m* is 128. Please note that Equation (5) is not the equation defined in [13] to compute the rank of node *n*, which is computed considering all nodes in the parent set. Instead, it computes the hypothetical rank that node *n* would have only considering the parent *i*.

The main procedure of the power planning strategy is defined in BuilDODAG (Algorithm 1). The procedure is executed until all nodes are connected to the DODAG (U=⌀).

The principle of the power planning algorithm consists of connecting the nodes to the DODAG in successive layers, which move radially away from the root at each round. The nodes in a layer closer to the root (inner layer) act as potential parents for nodes of the adjacent layer further from the root (outer layer). However, the order in which nodes are connected to DODAG is not only dependent on the Euclidean distance with respect to the root. The nodes of an inner layer may be poorly distributed around root, concentrated in a single region, and may not provide connectivity for the nodes of the adjacent outer layer. As a consequence, nodes further from the root, but closer to the nodes already connected, would be connected first. This effect is not desirable because the rank of the nodes would not represent its distance with respect to the root, resulting in a bad DODAG. To minimize the possibility of occurrence of this effect, an initial set of nodes, connected directly to the root must be selected to provide connectivity in all directions around the root. This initial set of nodes is computed by the InitDODAG procedure (Algorithm 2). This procedure selects at most *n* nodes that can connect directly to the sink (the set A), following a geometric approach that selects nodes in all directions around the root. As explained later in this section, the value of *n* is dependent on the geometric distribution of the nodes and is determined by an optimization procedure.

After initialization, the algorithm tries to connect one node at a time, by adjusting the power of the node and of its parents, if necessary. The node nearest to the root still not connected to the DODAG is evaluated first. If a suitable set of parents in C cannot be found, the node may jump to the next round. The jump procedure increases the possibility to select parents for the node because the number of nodes in C may be higher. The maximum number of jumps that a node may perform is controlled by α.
**Algorithm 1:** Power Planning Algorithm
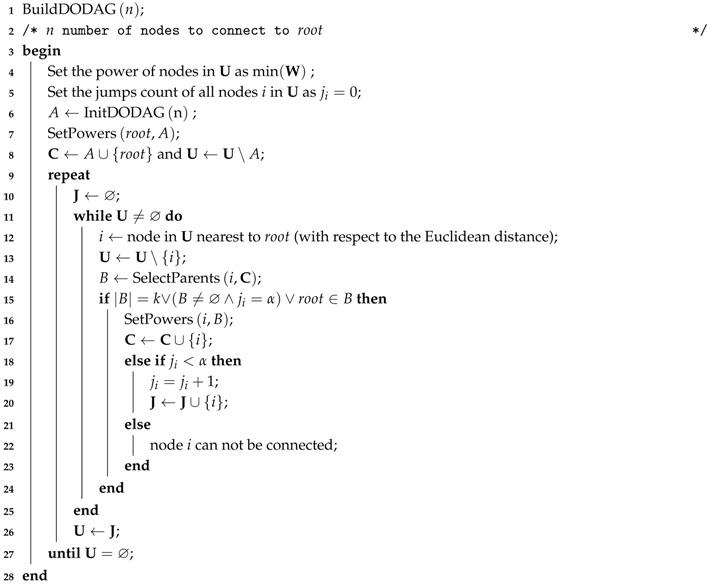

**Algorithm 2:** Select Nodes to Connect Directly to the Root
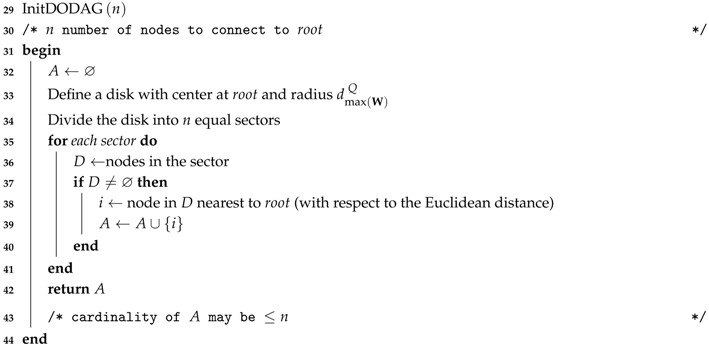


The SelectParents procedure (Algorithm 3) tries to find a suitable parent set for the new node considering the nodes already connected to the DODAG (the set C). A solution is considered suitable if the cardinality of the parent set returned by SelectParents is *k* or the node is connected directly to the sink. If this condition is not satisfied, the node may be included in the set J to “jump” to the next round. Nodes in J are re-evaluated after all nodes in U are tested.
**Algorithm 3:** Select the Parents to Connect a Node to the DODAG
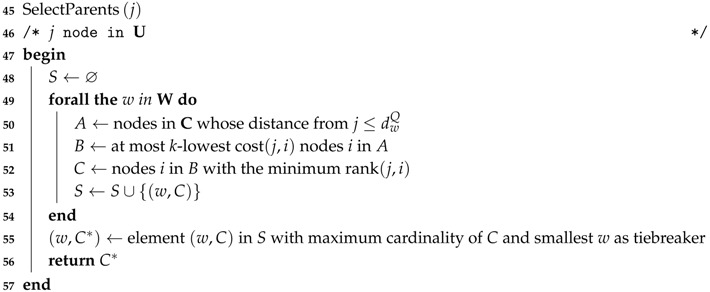


If a suitable solution is found, the transmission power of the node and of its parents is adjusted using SetPowers (Algorithm 4). This procedure adjusts the transmission power to assure that all links between the node and its parents have ETX≤Q. To calculate the value of *w* in step 3 of SetPowers, first it calculates the outage of the link O assuming $O=O_{ab}=O_{ba}$ and ETX=Q in Equation (3). Then, it computes $\bar{\gamma }$ from Equation (2). Finally, it computes *w* from $\bar{\gamma }=\frac{\eta_{d}\cdot w}{N_{0}\cdot B}$.
**Algorithm 4:** Adjust the Power of the Child and Parent Nodes
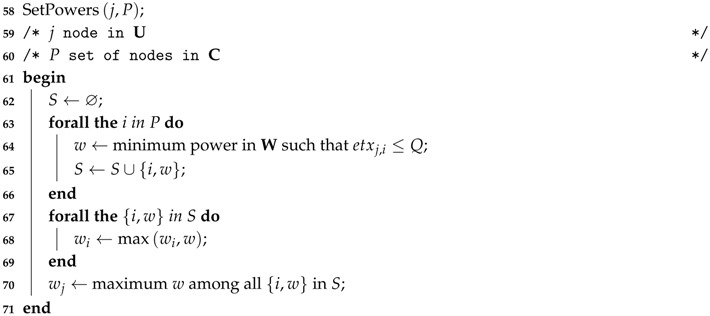


The worst-case time complexity of BuildDODAG is polynomial, and given by $T\left(N\right)=O\left(N^{2\alpha }\right)$, where *N* is the total number of nodes. BuildDODAG must receive the parameter *n* as input, corresponding to the initial set of nodes to connect to the root. The value of *n* is strongly dependent on the topology. The evaluation of random topologies has indicated that the same value of *n* may be inappropriate for networks with the same density. For this reason, *n* is defined by an optimization procedure that maximizes the following objective function:

(6) $$\left\{\mathrm{Floor}\left(\frac{AvgParentSet}{\theta }\right)*\theta ,-AvgPower\right\},$$

where AvgParentSet is the average parent set size considering all nodes except the root, and AvgPower is the average transmission power assigned to all nodes. The θ parameter is used to reduce the importance of small increments on the parent set size at the expense of an increase in the average power. The second element on the objective function is used only as a tiebreaker. This optimization problem is very simple because it is mono-variable and *n* is an integer.

### 4.1. Remarks on the Centralized Operation of the Algorithm

This section describes how the proposed algorithm could operate together with a centralized control application. The goal of the control application is to collect the necessary information for the algorithm execution and apply the produced transmission power setting. The principles described here are based on experiments conducted with the implementation of the 6LoWPAN-RPL stack that exists in the Contiki operating system [23].

The specification of 6LoWPAN [19] was produced by a working group of the IETF, aimed to optimize the use of IPv6 within low-speed wireless networks based on the IEEE 802.15.4 standard. Its main feature is a header compression mechanism to increase the amount of information (payload) that can be transmitted in a frame having a maximum transfer unit of 127 bytes. This is done by removing a number of fields in IPv6 and user datagram protocol (UDP) headers, either because they have known values, or because the values can be inferred from fields in the IEEE802.15.4 header. The 6LoWPAN border router (6LBR) supports the translation between regular IP and 6LoWPAN packets, and implements the functions of the DODAG root.

The control application uses the constrained application protocol (CoAP) to format the data and to ensure the delivery of control messages. CoAP is an application protocol designed specifically for the low power and lossy networks, providing an alternative to XML and HTTP [24]. CoAP uses a formatting style called representational state transfer (REST), which uses the same verbs of HTTP, such as GET, POST, PUT, and DELETE. The REST style uses uniform resource identifiers to interact with external objects, providing access through the verbs listed before.

**Figure 3:**
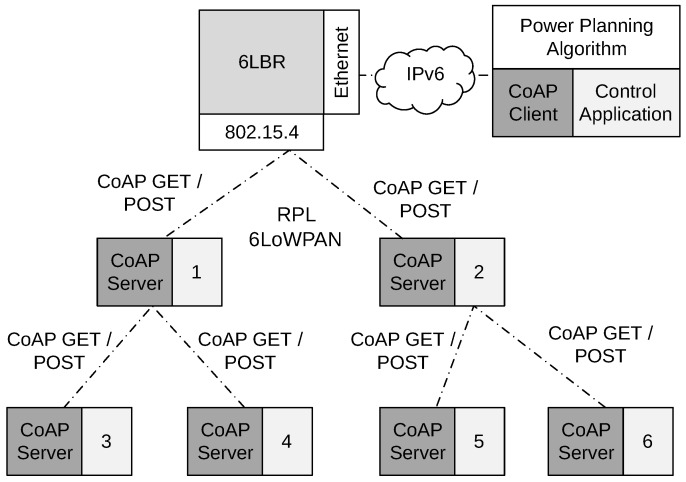
Centralized operation of the transmission power planning algorithm.

Figure 3 illustrates the principles of the centralized operation. One can observe an instance of 6LBR, provided with the Contiki platform. The control application runs outside the RPL-6LoWPAN network and communicates with 6LBR and with the AMI network nodes. Communication with 6LBR is necessary because it allows the control application to know what nodes are running at a given moment. The example shows an AMI network with six nodes, each one including the metering and communication functions. The communication function supports the exchange of messages between the metering devices and the utility, as well as communication between the nodes and the control application. The first is not shown in Figure 3, but the last is performed through CoAP messages. One can observe a CoAP client running with the control application and a CoAP server running inside the nodes.

The planning algorithm assumes to know the location of each node. Because the AMI network is a structured wireless network, location information is pre-configured in each node before field deployment and collected by the control application during network operation. The algorithm computes an estimated initial value for ETX, which is configured in each node before starting network operation. In addition, the initial power configuration is computed offline and it is stored in the nodes before the deployment. When the nodes begin to operate, RPL messages are exchanged, and the 6LBR becomes aware of active nodes. When 6LBR identifies a node, it reports to the control application, which then uses GET messages to read its location, neighbors and ETX. The GET messages include a registration to observe the resources (neighbors and ETX). In the CoAP terminology, the client retrieves a representation of the resources and requests the server to update this representation. When a node starts unicast communication, the MAC layer of Contiki updates the current neighbors and the quality of the links (ETX). The CoAP server notifies these resources periodically to the CoAP client, which, in turn, informs the control application. Every time a significant change occurs in the network topology, the control application restarts the power setting algorithm and uses POST messages to inform each node directly of the new transmission power value that it should use according to the new plan. Our method does not change RPL, only the transmission power of the nodes. When the topology changes because of link degradation or node failure, RPL uses its own mechanisms to reconfigure the network. However, due to our power planning mechanism, changes in topology will happen much less frequently.

## 5. Evaluation

In this section, we compare our planning strategy with two other approaches commonly used in the RPL experiments: fixed-power and vertex-based power assignment. The fixed-power strategy consists of assigning the same power to all nodes in the network. This simplistic approach is indeed the most used in the RPL experiments published in the literature. The vertex-based approach consists of adjusting the transmission power to assure that each node can communicate with a certain number of neighbors through links that satisfy an ETX-based quality constraint. Because our algorithm aims to improve RPL behaviour through transmission power planning, we identify it as DODAG-based.

In order to provide a fair comparison between methods, we assigned the same average power to the nodes on every evaluated method. The average power obtained with the DODAG-based planning is assigned to all nodes in the fixed approach. In the vertex-based approach, the number of neighbors is increased until the average power is the same as that obtained with the DODAG-based method. In some cases, imposing the power obtained with the DODAG-based planning may result in disconnected graphs for the fixed method and vertex-based method. We find the minimum increase of power that permits the connection of all nodes, by adjusting the power of the nearest nodes between unconnected sub-graphs.

The wireless channel conditions have been modeled using the Nakagami-*m* distribution and a log-distance path model. We assume that radios operate in the 914 MHz band. We have considered two distinct channel conditions: rural and urban areas. The path loss exponent (α) and the Nakagami fading severity (*m*) have been adjusted to match different channel conditions. In the scenario for rural areas, nodes were randomly placed within a disk of 1 km radius, and we have assumed α=2.5 and m=2. The radio power could range from –10 dBm to 10 dBm in steps of 2 dBm. For the urban area scenario, nodes have been distributed in a disk of radius 100 m, with α=3 and m=1. The transmission power could be adjusted from −12 dBm to 0 dBm in steps of 1 dBm. The root was placed in the mean position of all nodes, and all nodes are connected to the root using a single DODAG. To show that the proposed algorithm is capable of planning the transmission powers so that the ETX is low in both directions (i.e., the quality of the link is good), the maximum ETX imposed for links in rural and urban area conditions is 1.2. We pre-configure the ETX between each node and its neighbors by using the values calculated with Equation (3). Table 2 summarizes the evaluation parameters.

The proposed algorithm has two objectives. The first is to assure that the parent set satisfies a minimum predefined size, which allows for designing networks with a desired degree of resilience. The second is to assure that the links connecting nodes to their parents all have similar quality. This allows all nodes in the parent set to offer connectivity through paths of similar quality. Accordingly, we selected two metrics to evaluate the proposed algorithm: parent set size and the quality of the path through the preferred parent. The first is to evaluate if the power setting is able to achieve the resilience objective, and the second is to assess if, in addition to improving RPL resilience, the method does not worsen the quality of the preferred parent path. The performance of the method is evaluated by observing the two metrics jointly.

**Figure 4:**
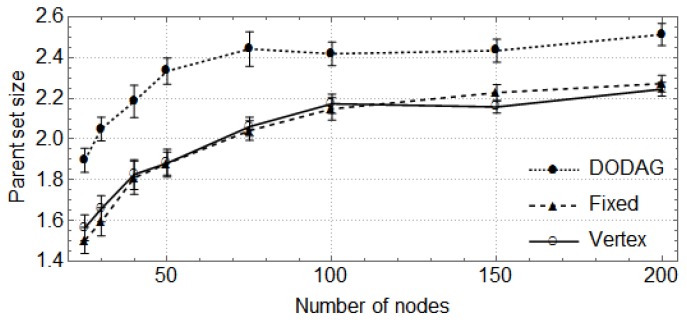
Parent set size in a rural area scenario.

**Figure 5:**
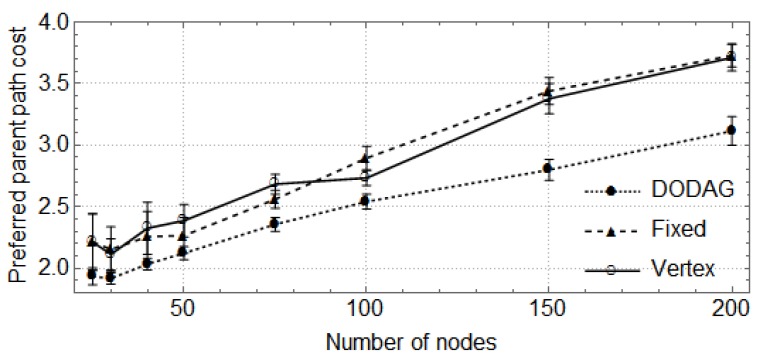
Preferred parent path cost in a rural area scenario.

**Figure 6:**
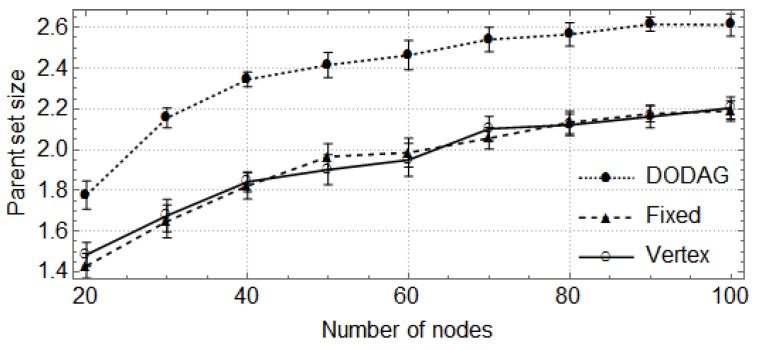
Parent set size in an urban area scenario.

**Figure 7:**
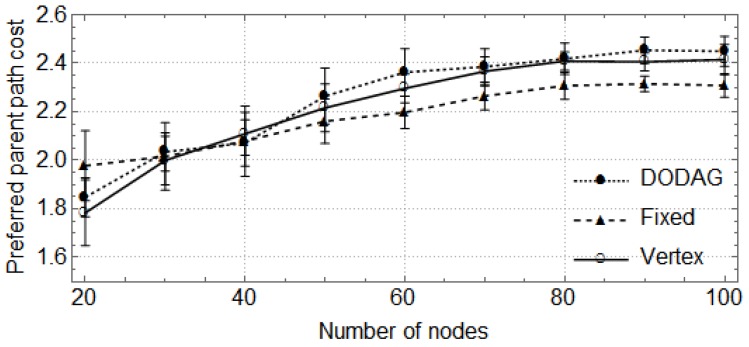
Preferred parent path cost in an urban area scenario.

**Figure 8:**
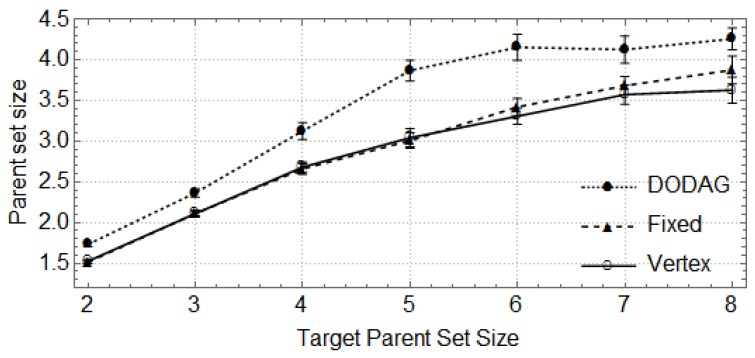
Parent set size *X* target parent set size in a rural area scenario.

**Figure 9:**
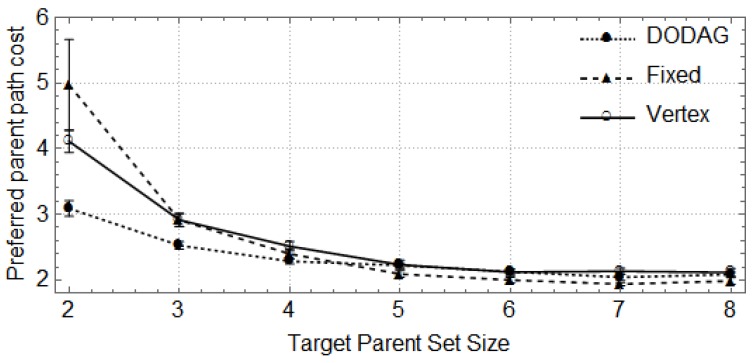
Preferred parent path cost *X* target parent set size in a rural area scenario.

In the following, the vertical bars represent the 0.95 confidence level of the average parent set size obtained with 30 random topologies. First, we show how the size of the network (number of nodes) affects the size of parent sets and the cost of paths through the preferred parent (Figure 4, Figure 5, Figure 6 and Figure 7). For these cases, the DODAG-based power planning algorithm was executed with a target parent set size k=3. Afterwards, we show how the target parent set size affects the same parameters for a network with 100 nodes in the rural scenario (Figure 8 and Figure 9).

**Figure 10:**
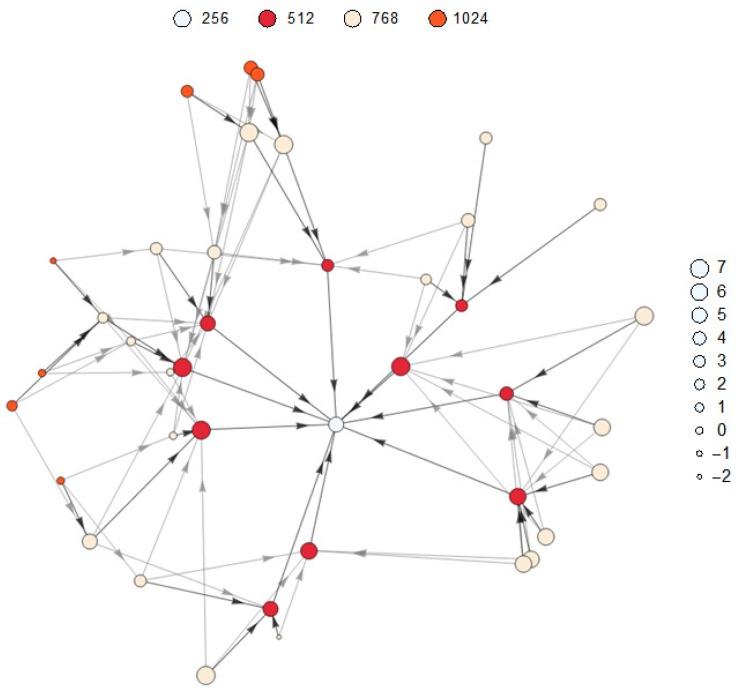
Power planning example.

The imposed number of parents k=3 is not achievable for all nodes because nodes that can communicate directly to the root have only one parent. This can be observed in the example shown in Figure 10, which represents the DODAG obtained after the convergence of the RPL. In the figure, the size of the nodes represents the transmission power (in dBm) and the color represents the rank. The darker lines connect the child to the preferred parent while the lighter lines to the other parents in the parent set. The nodes with rank equal to 512 connect directly to the root. All links represented in the graph satisfy the restriction imposed on ETX.

The results for the rural area scenario are shown in Figure 4 and Figure 5. In Figure 4, one observes that the sizes of parent sets obtained by the DODAG method are closer to the target than those obtained by the other methods. For networks with up to 100 nodes, the DODAG approach reaches an average size of approximately 2.19, while the Vertex method reaches 1.79 and the Fixed method 1.77. For larger networks, the average size is 2.48 for the DODAG method and 2.21 for the other methods. The costs of the paths through preferred parents are shown in Figure 5. They are equivalent for the three methods when considering networks with up to 100 nodes, but, for larger networks, the DODAG approach overcomes the other methods, providing paths with costs 14.2% lower then the costs provided by Vertex and Fixed methods.

The results for the urban area scenario are shown in Figure 6 and Figure 7. In the first, we can observe that the sizes of parent sets obtained by the DODAG method are also closer to the target when compared to the other methods. For networks with up to 50 nodes, the average size obtained by the DODAG method is approximately 2.19, while the average size is 1.7 for the other methods. For larger networks, the average size is 2.57 for the DODAG approach and 2.11 for the other. The costs of the paths through preferred parents are presented in Figure 7. One can observe that the costs obtained by the DODAG method are slightly larger for networks with up to 50 nodes, but one can observe that the costs are statistically equivalent. For larger networks, the average costs obtained by the Fixed method are 5.6% lower when compared to the other methods.

If we take both metrics simultaneously in both scenarios, we can consider that the DODAG method reached the proposed objectives, since the sizes obtained for the sets of parents are always as close as possible to the target, while the costs obtained for the paths passing by the preferred parents are the best in the rural setting or very close to the best in the urban setting. Please note that the last scenario corresponds to dense networks where the algorithm has little room to act.

Figure 8 and Figure 9 show the effect of using different target values for parent set sizes (*k*) in a rural area scenario with 100 nodes. The DODAG method consistently outperforms the other methods with respect to the parent set size and also offers solutions with improved path cost for small values of *k*, as can be observed in Figure 4 and Figure 5. The actual parent set size obtained with RPL depends on the density of the network, and increasing *k* above a certain value has no effect. An important feature of the proposed algorithm is that the power assigned to the nodes is not increased if it does not improve the connectivity of the network.

## 6. Conclusions

This paper presented a transmission power planning method for DODAG topologies suitable to AMI networks. The study shows that it is possible to control the transmission power of nodes in RPL networks to provide more stable and resilient topologies. Without changing the protocol, the proposed method induces RPL built DODAGs where parent sets possess a minimum preset size, which allows the design of networks with a desired degree of resilience. The method also induces the construction of DODAGS where the links connecting nodes to their parents all have a similar quality, allowing the nodes in the parent set to offer connectivity through paths of similar quality. Besides the clear advantage of providing a more stable network, this approach improves the RPL performance because the rank of a node becomes a closer indication of the path cost to the root through this node.

Our algorithm has been evaluated in two distinct channel conditions: rural and urban areas. The findings show that the method is very promising because it is able to achieve the goals of resilience and stability and still maintain a good average cost of preferred paths in the urban setting, and better average costs in the rural setting. With respect to the latter aspect, the performance of our method is best for networks with poorly connected topology, which occurs when the distances between neighboring nodes are very variable. In this case, the quality of the links is worse and our algorithm has more room to make adjustments to improve results. For the rural scenario, which has this characteristic, it was shown that the algorithm is able to find paths through preferential parents with lower costs.

The interference between network nodes is not addressed in our study because this issue can be solved by assigning non-interfering transmission channels to nodes belonging to the same interference domain, i.e., using channels at distinct frequencies or non-overlapping time slots. However, to assure that the comparisons made in Section 5 are fair, we perform the evaluation with all methods spending the same amount of energy, thus subject to equivalent levels of interference. The interference of external resources is also not part of the scope of our study because this problem can be handled by the RPL, as discussed in the Section 1.

It should be noted that the mechanisms and algorithms presented can be used for other applications that require structured networks for which energy consumption is not a primary requirement. Many of the networks used in applications for smart cities may fit into this context.

In Section 4.1, we have provided comments on how the proposed algorithm could be used in a centralized communication architecture. In our future studies, we intend to specify an architecture based on SDN to support the centralized operation of the proposed algorithm. 

## Figures and Tables

**Table 1:** Symbols definition.

Symbol	Meaning
root	DODAG root
C	set of nodes connected to the DODAG
U	set of nodes not connected to the DODAG
W	discrete powers that can be assigned to the radio
$w_{i}$	current power of node *i*
*k*	scalar that controls the desired number of parents
$r_{i}$	current rank of node *i*
$etx_{n,i}$	ETX of the link between *n* and *i*
*m*	ETX multiplier
$d_{w}^{Q}$	maximum link length with power *w* and ETX≤Q
*h*	minimum hop rank increase
α	maximum allowable number of jumps
J	set of nodes that jumped in the iteration

**Table 2:** Evaluation parameters.

Parameter	Description	Value
Channel	Channel Type	Rural/Urban
*f*	Radio Operating Frequency	914 MHz
α	Path Loss Exponent	2.5 (rural)/3 9 (urban)
*m*	Nakagami-*m* Fading Severity	1 (urban)/2 (rural)
ETX	Maximum ETX	1.2
Area	Rural Area (disk radius)	1 km
Area	Urban Area (disk radius)	100 m
Power	Transmit Power Range (Rural)	−10 dBm to 10 dBm (2 dBm steps)
Power	Transmit Power Range (Urban)	−12 dBm to 0 dBm (1 dBm steps)
